# The Contributions of HIF-Target Genes to Tumor Growth in RCC

**DOI:** 10.1371/journal.pone.0080544

**Published:** 2013-11-18

**Authors:** Ting Zhang, Xiaohua Niu, Lili Liao, Eun-Ah Cho, Haifeng Yang

**Affiliations:** 1 Tianjin Institute of Urology, Second Hospital of Tianjin Medical University, Tianjin, China; 2 Department of Cancer Biology, Lerner Research Institute, Cleveland Clinic, Cleveland, Ohio, United States of America; 3 Department of Pathology, Anatomy and Cell Biology, Thomas Jefferson University, Philadelphia, Pennsylvania, United States of America; University of Hawaii Cancer Center, United States of America

## Abstract

Somatic mutations or loss of expression of tumor suppressor VHL happen in the vast majority of clear cell Renal Cell Carcinoma, and it’s causal for kidney cancer development. Without VHL, constitutively active transcription factor HIF is strongly oncogenic and is essential for tumor growth. However, the contribution of individual HIF-responsive genes to tumor growth is not well understood. In this study we examined the contribution of important HIF-responsive genes such as VEGF, CCND1, ANGPTL4, EGLN3, ENO2, GLUT1 and IGFBP3 to tumor growth in a xenograft model using immune-compromised nude mice. We found that the suppression of VEGF or CCND1 impaired tumor growth, suggesting that they are tumor-promoting genes. We further discovered that the lack of ANGPTL4, EGLN3 or ENO2 expression did not change tumor growth. Surprisingly, depletion of GLUT1 or IGFBP3 significantly increased tumor growth, suggesting that they have tumor-inhibitory functions. Depletion of IGFBP3 did not lead to obvious activation of IGFIR. Unexpectedly, the depletion of IGFIR protein led to significant increase of IGFBP3 at both the protein and mRNA levels. Concomitantly, the tumor growth was greatly impaired, suggesting that IGFBP3 might suppress tumor growth in an IGFIR-independent manner. In summary, although the overall transcriptional activity of HIF is strongly tumor-promoting, the expression of each individual HIF-responsive gene could either enhance, reduce or do nothing to the kidney cancer tumor growth.

## Introduction

The vast majority of renal cell carcinoma (RCC) cases are of the clear cell type. It is now known that the inactivation of the *VHL* tumor suppressor gene plays a causal role in the pathogenesis of clear cell renal cell carcinomas (ccRCC). In sporadic ccRCC tumors, about 70% of them harbor biallelic inactivation of *VHL* through mutation, deletion, or hypermethylation of promoter that leads to the loss of its expression [1]. In hereditary kidney cancer patients, the inherited germline mutation in one allele of *VHL* predisposes them to earlier onset bilateral kidney cancer. The protein product of *VHL* tumor suppressor protein, pVHL, is the substrate recognition unit of an E3 ubiquitin ligase complex that also contains Cul2, Elongin C and B, and Rbx1[2]. This complex targets the alpha subunits of the heterodimeric transcription factor HIF (Hypoxia-Inducible Factor) for ubiquitylation and destruction. There are three alpha subunits of HIF and for the simplicity they are referred to as HIFα. Under normoxia (normal oxygen tension), prolyl hydroxylase modifies HIFα on key proline residues (Pro) [3-5], which serve as a binding signal to the beta domain of pVHL. pVHL-containing complex then promotes ubiquitylation on HIFα, which leads to quick proteasomal degradation. Hypoxia (oxygen deprivation) or other pathological conditions prevents prolyl hydroxylation, and HIFα accumulates and forms complex with HIF1β. HIF complex binds to Hypoxic response element (HRE) and regulates transcription of HIF-responsive genes. Increased HIF activity as a result of *VHL* inactivation increases the expression of many genes and contributes to renal carcinoma growth. Notably, one of the genes whose expression is increased following VHL inactivation is VEGF, and VEGF and its receptor VEGFR are confirmed drug targets in ccRCC [6].

### Kidney cancer treatment and drug-resistance

Sunitinib (Sutent^®^) is a small molecule inhibitor of the receptor tyrosine kinases of the VEGF family [7], and it[8][9] is now the front-line standard of care in metastatic RCC. Other VEGFR inhibitors, such as sorafenib [10], axitinib [11] and pazopanib [12] were all reported to be active against ccRCC. However, about 20-30% of the patients do not respond to VEGFR inhibitor therapies and nearly all will become resistant [9]. These patients are in urgent need of new and effective therapies, and identifying new drug targets is a prerequisite step.

### Other HRGs other than VEGF are important for efficient tumor growth

The relative importance of constitutively active HIF pathway in kidney cancer initiation and maintenance has been well established in xenograft models [13,14]. Down-regulation of HIF2α expression in *VHL-/-* kidney cancer cells did not inhibit cellular growth under standard cell culture condition. However, it severely impaired these cells’ ability to form tumors in a xenograft model [15]. The transcriptional activity of the HIF2α was shown to be critical for its oncogenic activity [16,17], suggesting that the HIF-responsive genes were largely responsible for its ability to promote tumor growth. Consistent with this, Mxi-1, a c-Myc antagonist, was found to possess oncogenic activity [18]. Similarly, Oct4, a transcriptional factor essential for maintaining stem cell pluripotency [19], TGF-α, an agonist for EGFR [20], and Ror2, a receptor tyrosine kinase [21,22], were all shown to be induced by HIF2α and promoted tumor growth of kidney cancer cells. However, in addition to them, HIF regulates many aspects of cell biology such as cell cycle progression, metabolism and glucose homeostasis, and cell signaling. The contributions to tumor growth by HRGs involved in these processes were not fully explored, so in this study we studied the contributions to tumor growth by seven HRGs. We found that some HRGs enhanced tumor growth, some did nothing, while some were tumor-suppressive.

## Materials and Methods

### Cell culture

786-O kidney cancer cells with or without pCDNA3 based wild type HA-VHL were previously described [15]. The VHL status and the HIF activity were confirmed by anti-HA and anti-GLUT1 immunoblots. All the cell lines were maintained in glutamine-containing DMEM medium supplemented with 10% Fetal Bovine Serum (FBS) and 1% penicillin and streptomycin. For hypoxia mimetic treatment, either 200μM Deferoxamine (DFO, an iron chelator) or 20μM Cobalt Chloride (CoCl_2_, which replaces iron at the active site of the prolyl hydroxylases) was added to the cell culture media for twelve hours. The cells were washed and lysed for further analysis.

### Western Blot analysis

The cells were washed before being lysed with EBC buffer (50 mM Tris (pH 8), 120 mM NaCl, 0.5% NP-40). A protein assay kit (500-0006) from Bio-rad was used to determine the protein concentrations of the lysates. Samples with the same amount of total protein were boiled with sample buffer before being resolved by SDS-PAGE and analyzed with standard western blot techniques. The blots were developed with either Super Signal Pico substrate (Pierce Biotechnology, Rockford, IL) or Immobilon Western substrate (Millipore, Billerica, MA). Antibody against HIF1611079)waspurchased fromBDtransductionlaboratories (part of BD Biosciences, San Jose, CA). Antibodies against phopho-tyrosine PY-100 (9411), actin (4968), IGFIRβ (3018), and anti-Cyclin D1 antibody (2926) were from Cell Signaling Technology (Boston, MA). Anti-GLUT1 antibody (NB300-666) and anti-HIF2α (NB100-132) antibodies were purchased from Novus Biologicals (Littleton, CO). Anti-IGFBP3 (AF-675) was from R&D Systems (Minneapolis, MN). Antibodies against PARP1 (sc-7150), GAPDH (sc-59540) and Vinculin (sc-73614) were from Santa Cruz Biotechnology (Dallas, TX). 

### Short Hairpin RNAs (shRNAs)

shRNA constructs were obtained from Sigma. The sequences were listed in Table 1. 

**Table 1:** 

Name of shRNA constructs	shRNA sequences
HIF1b-1770	GAGAAGTCAGATGGTTTATTT
HIF2a-1631	CGACCTGAAGATTGAAGTGAT
HIF2a-566	CCATGAGGAGATTCGTGAGAA
VEGF-1137	AGGGCAGAATCATCACGAAGT
VEGF-1500	GCGCAAGAAATCCCGGTATAA
VEGF-1587	GACGTGTAAATGTTCCTGCAA
CCND1-326	GCCCTCGGTGTCCTACTTCAA
CCND1-539	CTCTAAGATGAAGGAGACCAT
CCND1-2322	GCCAGGATGATAAGTTCCTTT
ANGPTL4-786	GCAGAGTGGACTATTTGAAAT
ANGPTL4-1311	GAAGCTTAAGAAGGGAATCTT
EGLN3-692	GTGGCTTGCTATCCGGGAAAT
ENO2-1992	CGCCTGGCTAATAAGGCTTTA
ENO2-1735	CGCACTTTCCACTTCTTCCTT
GLUT1-1598	CCAAAGTGATAAGACACCCGA
GLUT1-455	GCGGAATTCAATGCTGATGAT
GLUT1-2310	GCCACACTATTACCATGAGAA
IGFBP3-633	CCTCCATTCAAAGATAATCAT
IGFBP3-681	CCAGCGCTACAAAGTTGACTA
IGFBP3-711	GAGCACAGATACCCAGAACTT
IGFBP3-770	GCCGTAGAGAAATGGAAGACA
IGFIR-532	GCGGTGTCCAATAACTACATT
IGFIR-1959	CGGCAACCTGAGTTACTACAT
IGFIR-3475	GCCGAAGATTTCACAGTCAAA
IGFIR-2427	GCCTTTCACATTGTACCGCAT

### Plasmids

Prolines 405 and 531 on pBabe-Puro-HA-tagged HIF2α-dPA were mutated to Alanines to escape VHL recognition. To destruct the transcriptional activity of the stabilized HIF2α, Proline 405 on pBabe-Puro-HA-tagged HIF2α-PA-dTA was mutated to Alanine, the amino acid residues 24-29 RCRRSK were mutated to ACAASA in the basic helix-loop-helix domain which would disrupt DNA binding, and the N-terminal and C-terminal transactivation domains (amino acid residues 450-572 and 820-870) were deleted. 

### VEGF ELISA

Protein concentration of VEGF was measured by an Elisa kit (Human VEGF Quantikine ELISA kit; R&D Systems) according to the manufacturer’s instruction. Briefly, 200μl of standard, control or sample was added to each well and incubated for 2 hours. After three washes, 200μl of VEGF conjugate was added for a further 2 hours. After washing, 200μl of substrate solution was added to each well. After twenty minutes incubation, the reaction was stopped with 50μl stop solution and the absorbance was read at 450 nm. The experiments were performed in duplicates.

### Real-time RT-PCR

Total RNA was extracted from cells with Trizol reagent (Invitrogen) following the instructions from the manufacturer. RNA concentration was determined by absorbance at 260nm. A First-strand cDNA Synthesis kit was used to generate First-strand cDNA (Origene). qRT-PCR was performed using the 7500HT Fast Real-time PCR System (Applied Biosystems) or with RT^2^ Real-Time^™^ ROX PCR Master Mix from SABiosciences. Genes were amplified using the primers described in Table S1. All quantifications were normalized to β-actin. 

### In vitro proliferation assays

In vitro cell proliferation assays were performed using a Cell Proliferation Kit II (XTT) from Roche Diagnostics following the manufacturer's instructions. 

### Nude mouse xenograft assays and statistical analysis

All animal works were performed with strict accordance with the recommendations in the Guide for the Care and Use of Laboratory Animals of the National Institute of Health. The protocol was approved by a Cleveland Clinic Institutional Animal Care and Use Committee (ARC 08850). The mice were anesthetized with isoflurane before subcutaneous injection of cancer cells, and all efforts were made to minimize pain and suffering. Subcutaneous nude mice xenograft assays were performed as previously described [13]. 10^7^ viable cells of a cell line were injected subcutaneously into one flank of a nude mouse, and the same number of cells of another cell line was injected into another flank of the same nude mouse. For each comparison ten mice were injected in two batches. The mice were sacrificed 8 to 10 weeks after injection, and tumors were excised and weighed. Results are presented as mean ± standard error of the mean. Results were evaluated statistically using Mann-Whitney U statistic analysis or t-test from SigmaPlot.

## Results

### A functional HIF is essential for tumor growth by VHL-deficient kidney cancer cells

**Figure 1:**
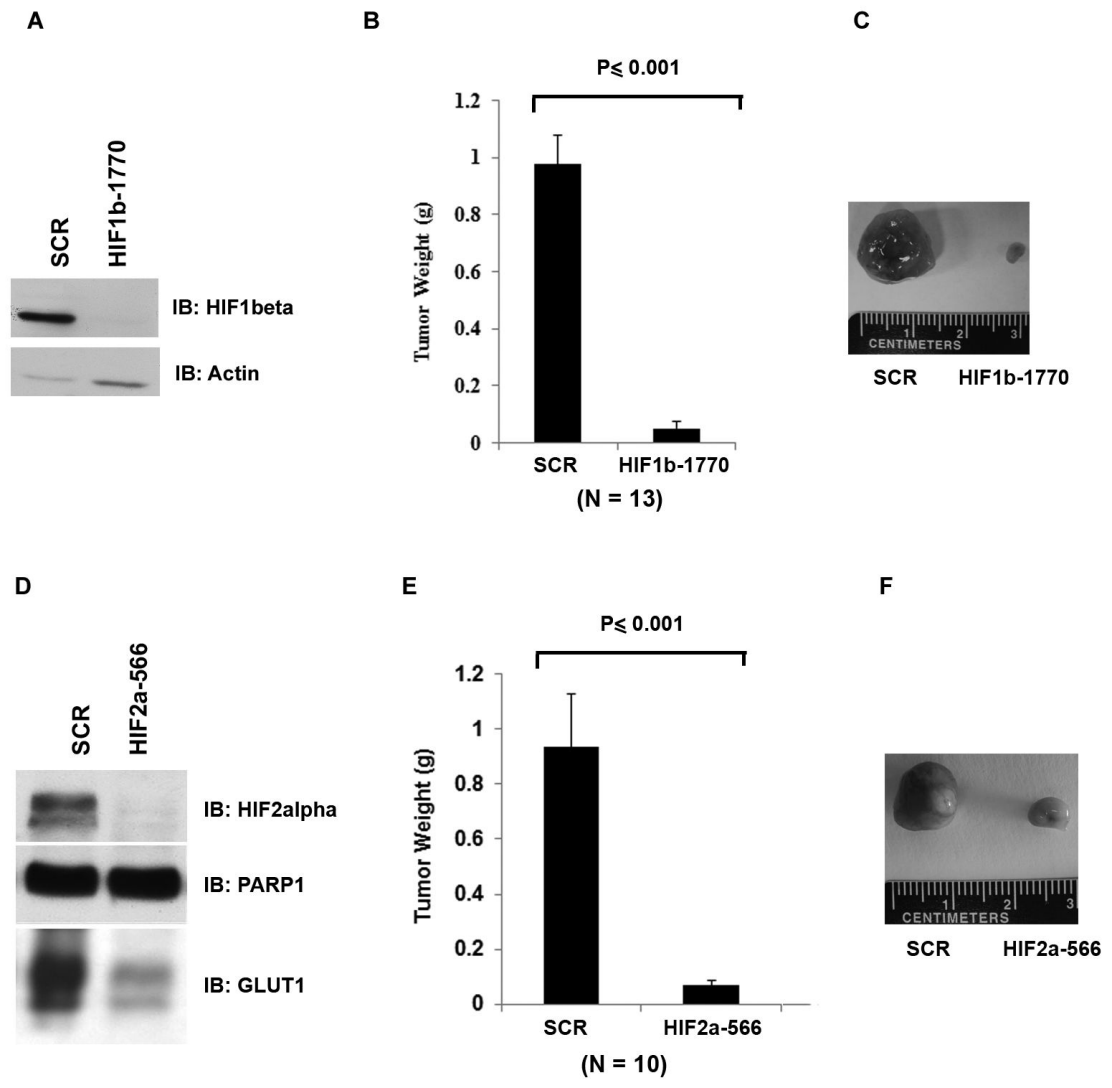
HIF is essential to tumor growth by VHL-deficient RCC cells. A. Total cell lysates of human renal carcinoma 786-O cells stably expressing the control shRNA (SCR) or the HIF1b-1770 were prepared and immunoblotted with the indicated antibodies. B. 786-O *VHL-/*- cells infected to produce SCR or HIF1b-1770 were injected subcutaneously into the flanks of nude mice. Approximately 8-10 weeks later, tumors were excised and weighed. Thirteen tumors per line were analyzed. Error bars = standard error of the mean. P<=0.001 according to a Mann-Whitney U statistic analysis. C. Representative photographs of tumors analyzed in Figure 1B. Left: Tumor from cells expressing SCR; Right: Tumor from cells expressing HIF1b-1770. D. Total cell lysates of 786-O cells stably expressing the SCR or the HIF2a-566 were immunoblotted with the indicated antibodies. E. 786-O *VHL-/*- cells infected to produce SCR or HIF2a-566 were injected subcutaneously into the flanks of nude mice. Approximately 8-10 weeks later, tumors were excised and weighed. Ten tumors per line were analyzed. Error bars = standard error of the mean. P<=0.001 according to a Mann-Whitney U statistic analysis. F. Representative photographs of tumors analyzed in Figure 1E. Left: Tumor from cells expressing SCR; Right: Tumor from cells expressing HIF2a-566.

The transcriptional factor Hypoxia Inducible Factor (HIF) is constituted of two subunits: the labile HIFα and the stable HIFβ. pVHL targets HIFα for degradation, so HIFα protein stabilizes and becomes constitutively active in VHL-defective ccRCC cancer cells [2]. The absence of VHL, which leads to high level of HIF2α, did not affect the cell growth rates in petri dishes (Figure S1A in File S1). However, high-level expression of HIF2α protein has been shown to be necessary and sufficient for 786-O ccRCC cancer cells to grow into tumors in orthotopic and subcutaneous xenograft mouse models [13,15-17,23]. To reconfirm the importance of HIF to tumor growth in a xenograft model, the expression of HIF1β protein in 786-O cells was stably suppressed by an shRNA construct HIF1b-1770 (Figure 1A). Suppression of HIF1β’s expression in 786-O cells also eliminated the expression of GLUT1, a marker of HIF’s transcriptional activity (Figure S2 in File S1). As expected, the inhibition of HIF1β expression significantly impaired the tumor growth by 786-O cells when compared to the same cells carrying an shRNA construct expressing a control sequence that does not target any known gene (SCR) (Figure 1B and C). Consistent with the previously reported importance of HIF2α to tumor growth [15], suppression of the other HIF subunit, HIF2α, also decreased GLUT1 expression and significantly impaired the tumor growth (Figure 1D, E and F).

### Validation of HIF-target genes in 786-O cells

**Figure 2:**
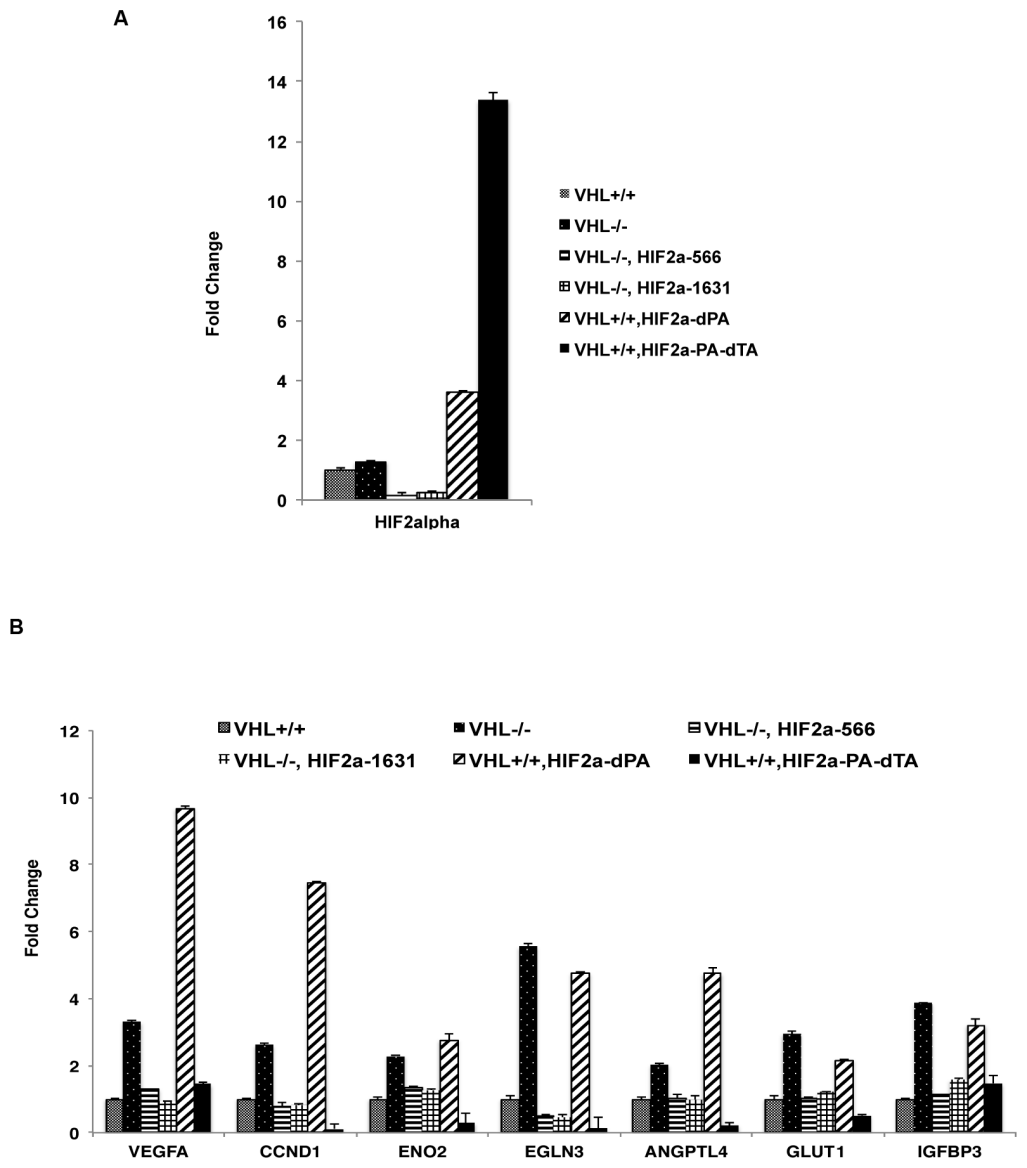
Validation of HIF target genes in VHL-deficient RCC cells. A. The mRNA levels of HIF2α in 786-O *VHL+/+* and *VHL-/*- cells expressing indicated plasmids or shRNA constructs were measured with real-time PCR. B. The mRNA levels of HIF target genes in 786-O *VHL+/+* and *VHL-/*- cells expressing indicated plasmids or shRNA constructs were measured with real-time PCR.

In order to examine the relative contributions of individual HIF-responsive genes (HRG) to the tumor growth, we wished to validate the HRGs that are tightly regulated by HIF’s transcriptional activity in 786-O cells before choosing the targets among them. In *VHL-/-* cells, the expression of a HRG should be significantly reduced after HIF2α expression is suppressed by shRNA. In *VHL+/+* cells (where HA-VHL was stably expressed in the *VHL-/-* cells), HRG’s expression should be significantly enhanced by a stable and functional HIF2α mutant but not by an inactive one. We confirmed that two independent HIF2α shRNA constructs significantly reduced HIF2α mRNA in *VHL-/-* cells, and the *VHL+/+* cells with stably integrated plasmids expressing HIF2a-dPA (active) and the HIF2a-PA-dTA (inactive) mutants greatly increased the total HIF2α mRNA levels (Figure 2A). 

Next the total RNA was extracted from these cells and real-time PCR analysis was performed to analyze the expressions of potentially interesting HRGs previously identified through microarray analysis. Many genes were confirmed to be bono fide HRGs that are tightly regulated by HIF’s transcriptional activity (Figure 2B and Figure S3 in File S1) while some were not robustly induced by HIF (data not shown). Among the many validated HRGs, we decided to further analyze the contributions to tumor growth by VEGF, Cyclin D1 (CCND1), ENOLASE2 (ENO2), EGLN3, ANGPTL4, GLUT1, and IGFBP3, because they are involved in many well-known aspects of HIF biology but their roles were not directly investigated in a ccRCC xenograft model. VEGF and VEGF receptors are the most important targets in current clinical treatment of RCC [6]. ANGPTL4 is thought to play critical roles in cancer growth and progression, angiogenesis, and metastasis[24]. Cyclin D1 is a very important cell cycle regulator and is reported to be oncogenic in breast cancer [25], and it was also reported to be a faithful target of HIF2α that might play an important role in kidney cancer [26]. ENO2 converts 2-phospho-D-glycerate into phosphoenolpyruvate and is a key component of the glycolytic pathway that is critical to ENO1-deficient glioblastoma cancer cells [27]. EGLN3 is one of the most induced HRGs that could hydroxylate the most critical proline residues on the HIFα proteins and have HIF-independent targets [28-31]. GLUT1 is a key glucose transporter that imports glucose into cancer cells [32]. IGFBP3 is an important regulator of Insulin-like growth factor pathway [33].

### VEGF and Cyclin D1 both positively contribute to tumor growth

**Figure 3:**
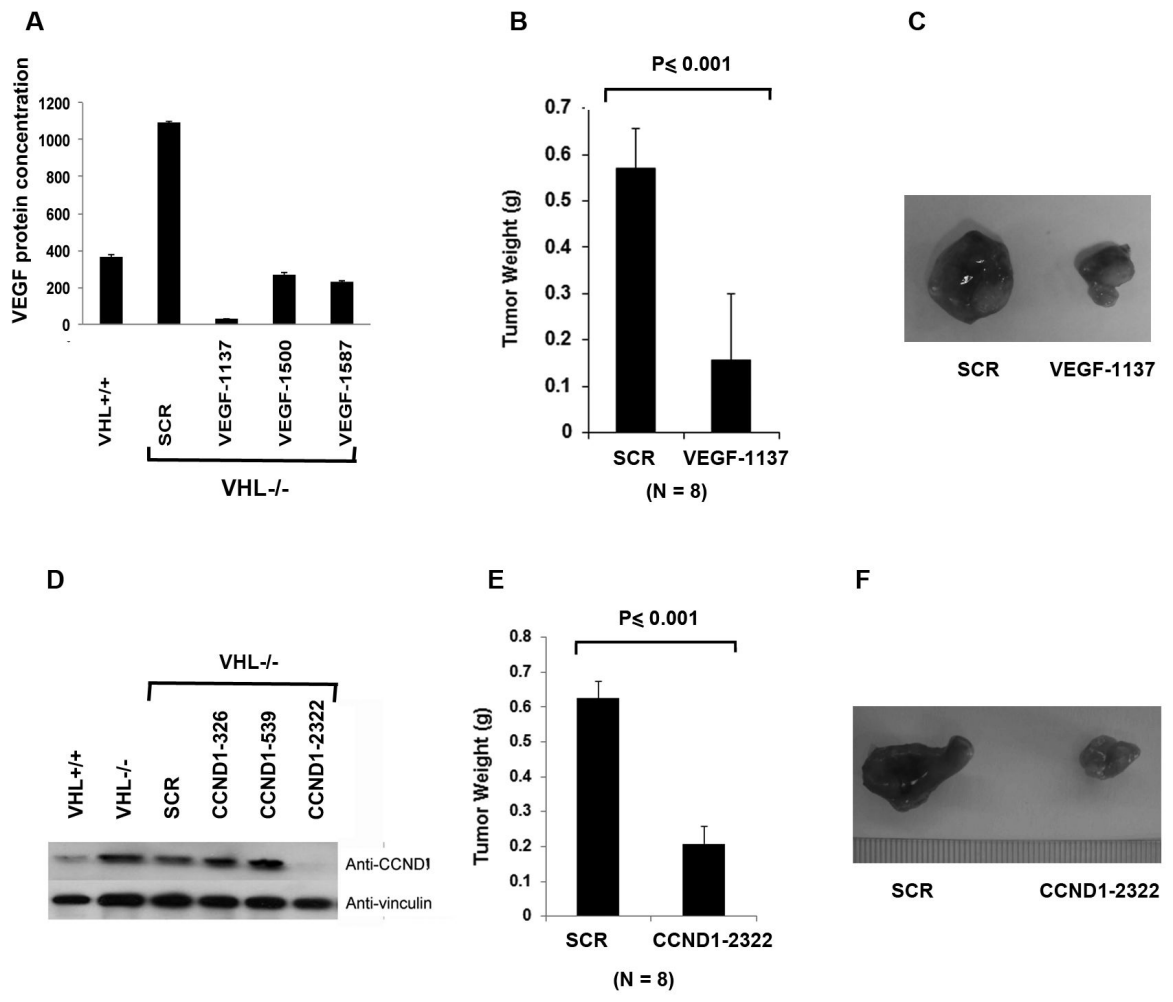
VEGF and Cyclin D1 positively contribute to tumor growth by VHL-deficient RCC cells. A. Lysates from 786-O cells stably expressing VHL, the SCR or the VEGF shRNAs were prepared and assayed with VEGF ELISA. B. 786-O *VHL-/*- cells infected to produce SCR or VEGF-1137 were injected subcutaneously into the flanks of nude mice. Approximately 8-10 weeks later, tumors were excised and weighed. Eight tumors per line were analyzed. Error bars = standard error of the mean. P<=0.001 according to a Mann-Whitney U statistic analysis. C. Representative photographs of tumors analyzed in Figure 3B. Left: Tumor from cells expressing SCR; Right: Tumor from cells expressing VEGF-1137. D. Total cell lysates of 786-O cells stably expressing VHL, the SCR or the Cyclin D1 shRNAs were immunoblotted with the indicated antibodies. E. 786-O *VHL-/*- cells infected to produce SCR or CCND1-2322 were injected subcutaneously into the flanks of nude mice. Approximately 8-10 weeks later, tumors were excised and weighed. Eight tumors per line were analyzed. Error bars = standard error of the mean. P<=0.001 according to a t-test. F. Representative photographs of tumors analyzed in Figure 3E. Left: Tumor from cells expressing SCR; Right: Tumor from cells expressing CCND1-2322.

VEGF is a potent pro-angiogenic factor that is critical for tumor-induced neo-angiogenesis and the current frontline anti-cancer drugs such as sunitinib, pazopanib and sorafinib all inhibits VEGF receptors and they have clearly shown positive clinical efficacy [6]. However, although sunitinib is generally very effective in shrinking tumors, the xenograft tumors formed by the 786-O *VHL-/-* cell line tend to be only mildly responsive to sunitinib treatment in mice [34,35], thus it raises the question whether VEGF is critical for 786-O *VHL-/-* cells to grow into tumor efficiently in mice. To address this, we identified an shRNA construct VEGF-1137 that very efficiently suppressed the VEGF expression below the level seen in the *VHL+/+* cells (Figure 3A). Like shRNA constructs against HIF, VEGF-1137 did not significantly change the cell growth in the petri dish (Figure S1B in File S1). We then compared the tumor growth of *VHL-/-* 786-O cells expressing a control shRNA (SCR) with that of the same cells expressing VEGF-1137. The cells depleted of VEGF generated much smaller tumors than the control cells in the same mice (Figure 3B and C), suggesting that HIF-induced VEGF expression contributed positively to the tumor growth by *VHL-/-* cancer cells.

Cyclin D1 is a cell cycle regulator that promotes cell proliferation along with many other important functions that enhances cancer development [36]. It is also another HRG that has been shown to play an important oncogenic role in breast cancer [25]. As it is a faithful target for HIF2α in RCC cells [26], it might also have a significant impact on RCC biology. To investigate its role in tumor growth of kidney cancer, its expression in *VHL-/-* cells were very efficiently knocked down by CCND1-2322 (Figure 3D). CCND1-2322 did not significantly change the cell growth in petri dish (Figure S1C in File S1). However, these cells grew into much smaller tumors than the cells expressing SCR (Figure 3E and F), which strongly suggests that Cyclin D1, like VEGF, also promoted tumor growth by the *VHL-/-* cancer cells in mice.

### Suppression of neither ANGPTL4, EGLN3, nor ENOLASE 2 significantly change the tumor growth

**Figure 4:**
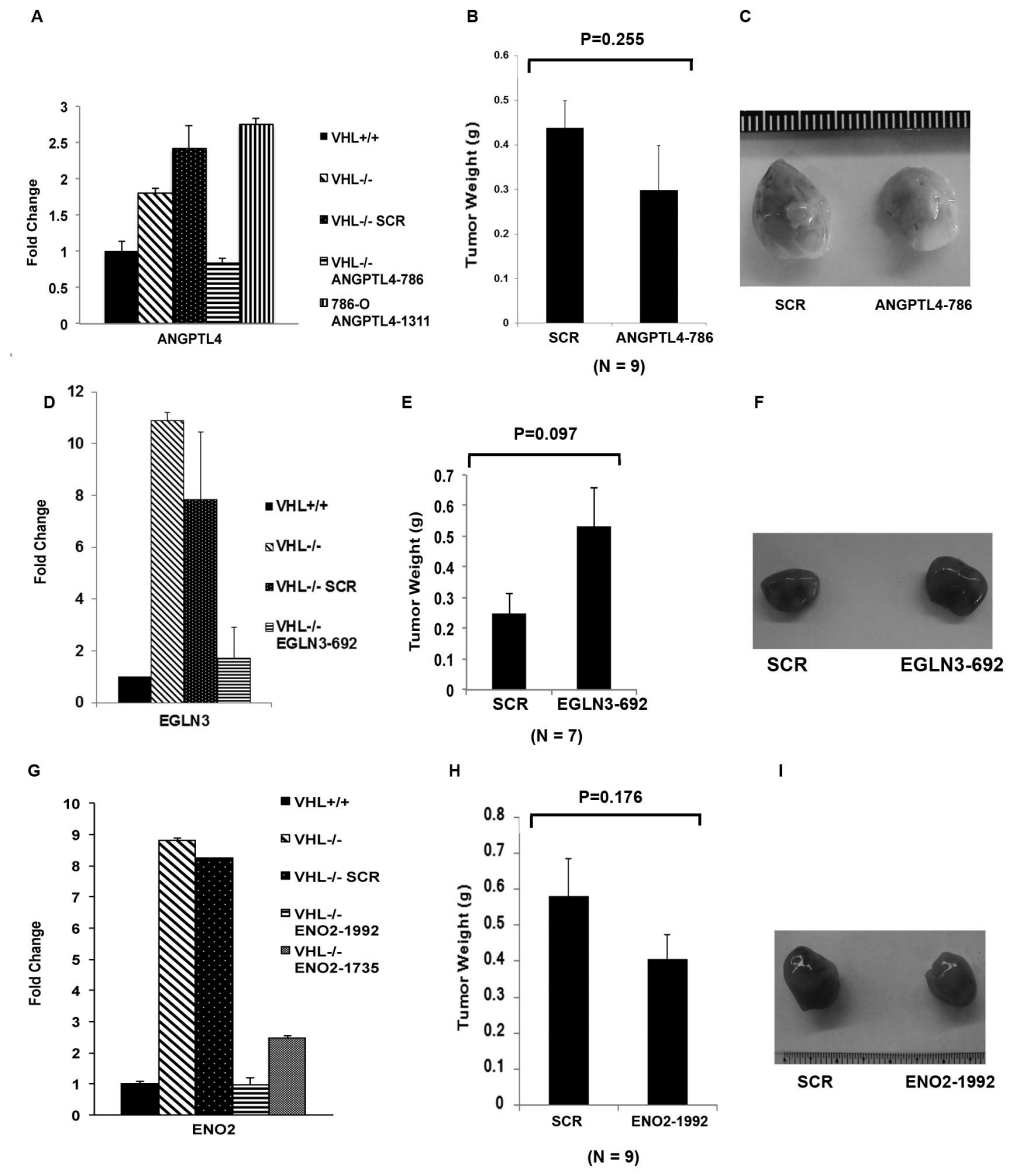
ENO2, EGLN3, and ANGPTL4 make no significant contribution to tumor growth by VHL-deficient RCC cells. A. The mRNA levels of ANGPTL4 in 786-O cells stably expressing VHL, the SCR or the ANGPTL4 shRNAs were analyzed with real-time PCR. B. 786-O *VHL-/*- cells infected to produce SCR or ANGPTL4-786 were injected subcutaneously into the flanks of nude mice. Approximately 8-10 weeks later, tumors were excised and weighed. Nine tumors per line were analyzed. Error bars = standard error of the mean. P=0.255 according to a t-test. C. Representative photographs of tumors analyzed in Figure 4B. Left: Tumor from cells expressing SCR; Right: Tumor from cells expressing ANGPTL4-786. D. The mRNA levels of EGLN3 in 786-O cells stably expressing VHL, the SCR or the EGLN3 shRNA were analyzed with real-time PCR. E. 786-O *VHL-/*- cells infected to produce SCR or EGLN3-692 were injected subcutaneously into the flanks of nude mice. Approximately 8-10 weeks later, tumors were excised and weighed. Seven tumors per line were analyzed. Error bars = standard error of the mean. P=0.097 according to a Mann-Whitney U statistic analysis. F. Representative photographs of tumors analyzed in Figure 4E. Left: Tumor from cells expressing SCR; Right: Tumor from cells expressing EGLN3-692. G. The mRNA levels of ENO2 in 786-O cells stably expressing VHL, the SCR or the ENO2 shRNAs were analyzed with real-time PCR. H. 786-O *VHL-/*- cells infected to produce SCR or ENO2-1992 were injected subcutaneously into the flanks of nude mice. Approximately 8-10 weeks later, tumors were excised and weighed. Nine tumors per line were analyzed. Error bars = standard error of the mean. P=0.176 according to a t-test. I. Representative photographs of tumors analyzed in Figure 4H. Left: Tumor from cells expressing SCR; Right: Tumor from cells expressing ENO2-1992.

Recent discoveries revealed that ANGPTL4 was involved in angiogenesis, altered redox regulation, and metastasis [24], so we decided to investigate its role in kidney cancer progression. The mRNA level of ANGPTL4 in *VHL-/-* cells was twice as much as that in *VHL+/+* cells, and the shRNA construct ANGPTL4-786 canceled that increase in the *VHL-/-* cells (Figure 4A). ANGPTL4-786 did not significantly change the cell growth in petri dish (Figure S1D in File S1). ANGPTL4-786 also failed to change the tumor growth by *VHL-/-* cells significantly (Figure 4B and C).

Next we examined whether EGLN3, one of the most highly induced HRG [17,28], plays any role in tumor growth. Although normally EGLN1 is the major proline hydroxylase for HIFα proteins, under certain conditions EGLN3 could also modify HIFα proteins for proteasomal degradation [37,38]. 786-O cells have no pVHL, so even if HIFα is proline hydroxylated it would not affect its protein stability. EGLN3 also was also reported to have non-HIF substrates and possess HIF-independent biological activities [29-31]. Surprisingly, the shRNA construct EGLN3-692 that efficient suppressed the protein expression of EGLN3 did not reduce the tumor growth (the small increase in tumor weight after EGLN3 knockdown was not statistically significant) (Figure 4D, E, and F). EGLN3-692 did not significantly change the cell growth in petri dish either (Figure S1E in File S1).

Enolase 2 (ENO2) is an enzyme that hydrolyses 2-phospho-D-glycerate into phosphoenolpyruvate, a critical step in the glycolysis pathway that converts glucose into pyruvate and generates ATPs without using molecular oxygen. It was reported that in VHL-defective kidney cancer cells the elevated HIF activity caused the decrease of the amount and repression of the function of mitochondria and shifted the cellular metabolism to glycolysis [39], so we wanted to examine the contribution of ENO2, which is critical to glycolysis, to tumor growth. The mRNA level of ENO2 was much higher in *VHL-/-* cells than that in *VHL+/+* cells, and the ENO2-1992 shRNA construct efficiently reduced its expression in *VHL-/-* cells (Figure 4G), but it did not significantly change the cell growth in petri dish (Figure S1F in File S1). We found that ENO2-1992 failed to change tumor growth significantly (Figure 4H and I). 

### Suppression of HIF-induced genes GLUT1 or IGFBP3 strongly enhance tumor growth

**Figure 5:**
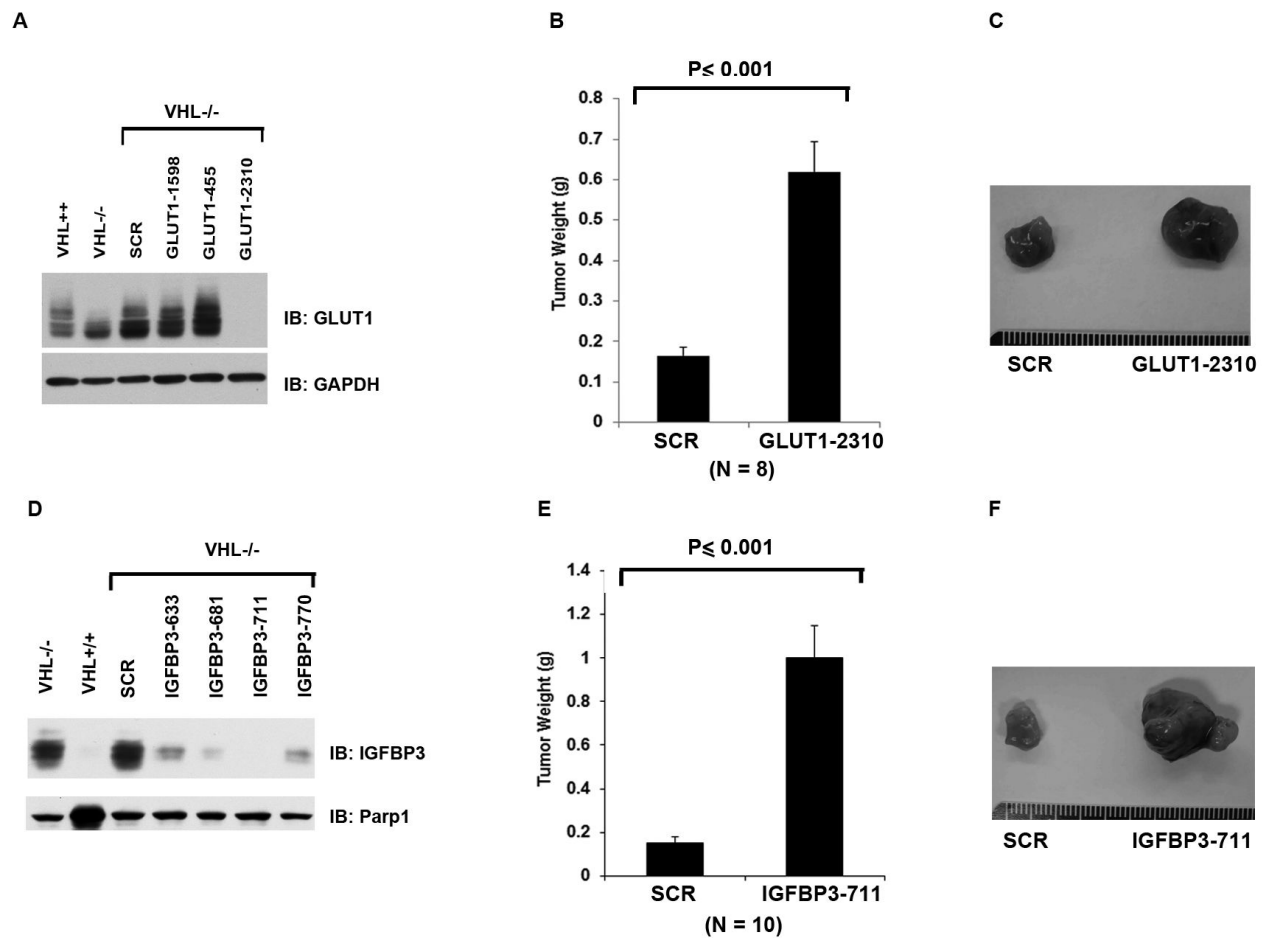
GLUT1 and IGFBP3 are tumor suppressive HRGs in 786-O cells. A. Lysates from 786-O cells stably expressing VHL, the SCR or the GLUT1 shRNAs were prepared and immunoblotted with indicated antibodies. B. 786-O *VHL-/*- cells infected to produce SCR or GLUT1-2310 were injected subcutaneously into the flanks of nude mice. Approximately 8-10 weeks later, tumors were excised and weighed. Eight tumors per line were analyzed. Error bars = standard error of the mean. P<=0.001 according to a Mann-Whitney U statistic analysis. C. Representative photographs of tumors analyzed in Figure 5B. Left: Tumor from cells expressing SCR; Right: Tumor from cells expressing GLUT1-2310. D. Total cell lysates of 786-O cells stably expressing VHL, the SCR or the IGFBP3 shRNAs were immunoblotted with the indicated antibodies. E. 786-O *VHL-/*- cells infected to produce SCR or IGFBP3-711 were injected subcutaneously into the flanks of nude mice. Approximately 8-10 weeks later, tumors were excised and weighed. Ten tumors per line were analyzed. Error bars = standard error of the mean. P<=0.001 according to a Mann-Whitney U statistic analysis. F. Representative photographs of tumors analyzed in Figure 5E. Left: Tumor from cells expressing SCR; Right: Tumor from cells expressing IGFBP3-711.

The constitutively high level of HIF activity shifts the VHL-defective kidney cancer cells metabolism from oxidative phosphorylation toward glycolysis to derive ATP without using molecular oxygen [39]. However, the efficiency of ATP generation through glycolysis is much lower than oxidative phosphorylation, thus the cancer cells demand for glucose is higher than that of the normal cells. To achieve that, HIF strongly induces the expression of GLUT1, a glucose transporter that enables the cancer cells to import glucose efficiently. To address the importance of GLUT1 to tumor growth, we successfully eliminated the GLUT1 expression by stably expressing GLUT1-2310 in these cells (Figure 5A). GLUT1-2310 did not significantly change the cell growth in petri dish (Figure S1G in File S1). Surprisingly and contrary to the hypothesis that GLUT1 was essential to tumor growth, its loss actually increased tumor growth (Figure 5B and C). The possible reasons for this observation will be discussed later.

IGFBP3 is another HRG that is highly induced by HIF and it is known to have IGF-dependent and IGF-independent biological functions that are important for cancer development [33]. To examine its contribution to tumor growth, we identified several shRNA constructs that suppressed IGFBP3 protein expression in *VHL-/-* cells (Figure 5D). IGFBP3-711 was chosen for further analysis because it was the most effective construct. IGFBP3-711 did not significantly change the cell growth in petri dish (Figure S1H in File S1). Interestingly, suppression of IGFBP3 expression, instead of retarding tumor growth, very significantly enhanced the tumor growth (Figure 5E and F).

### IGF1R is tumor-promoting in 786-O *VHL-/*- cells

IGFBP3 belongs to a family of proteins that bind to insulin like growth factors and either inhibit or stimulate the IGF receptors. In order to investigate whether IGFIR activity is affected by IGFBP3 depletion in 786-O cells, we probed the tyrosine phosphorylation signals on IGFIR. We found that depletion of IGFBP3 did not visibly change the tyrosine phosphorylation signals associated with IGFIR, suggesting that there is little change of IGFIR activity (Figure S4 in File S1). 

**Figure 6:**
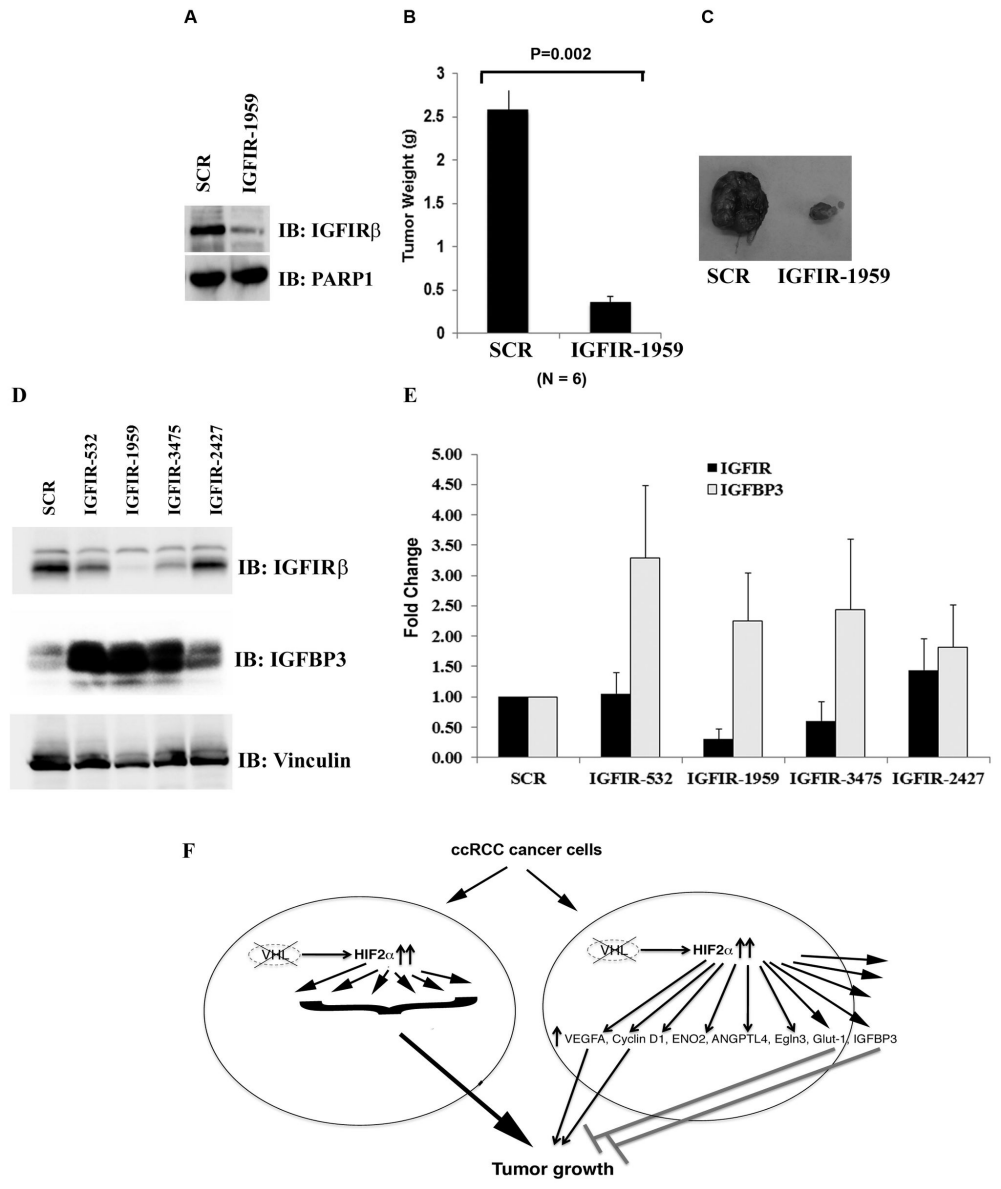
IGFIR loss suppresses tumor growth by *VHL*-defective RCC cells. A. Lysates from 786-O *VHL-/*- cells expressing SCR or an IGFIR shRNA were immunoblotted with indicated antibodies. B. 786-O *VHL-/*- cells infected to produce the SCR or IGFIR-1959 were injected subcutaneously into the flanks of nude mice. Approximately 9 weeks later, tumors were excised and weighed. Six tumors per line were analyzed. Error bars = standard error of the mean. P=0.002 according to a Mann-Whitney U statistic analysis. C. Representative photographs of nude mice and tumors analyzed in Figure 6B. Left: Tumor from cells expressing SCR; Right: Tumor from cells expressing IGFIR-1959. D. Lysates from 786-O *VHL-/*- cells expressing SCR or IGFIR shRNAs were immunoblotted with indicated antibodies. E. The mRNA levels of IGFIR and IGFBP3 in 786-O *VHL-/*- cells expressing SCR or IGFIR shRNAs were analyzed with real-time PCR. F. A model depicting how HIF and HRGs regulate tumor growth in ccRCC.

We then sought to examine whether IGFIR plays an oncogenic role in our model. Indeed IGFIR-1959 that effectively reduced IGFIRβ expression very significantly reduced tumor growth (Figure 6A, B and C). When we investigated the mutual interaction between IGFIR and IGFBP3, we made a surprising discovery that IGFIRβ suppression led to profound up-regulation of the protein levels of IGFBP3 (Figure 6D). As IGFIR-532, IGFIR-1959, and IGFIR-3475 reduced IGFIR protein expression to various extents, and all of them caused IGFBP3 up-regulation, this was unlikely to be an off-target side effect of a specific shRNA construct. The measurement of mRNA levels of IGFIR and IGFBP3 further revealed that IGFIR loss caused the increase of IGFBP3 mRNA levels, which at least partially explained the increases of IGFBP3 protein levels (Figure 6E). It remains a distinct possibility that the strong tumor suppressive effect of IGFIR loss was mostly due to the high levels of IGFBP3. If this is the case, since IGFIR is depleted, IGFBP3 must act in an IGFIR-independent manner.

## Discussion

Although VEGF and VEGFR have been proved to be critical targets for RCC treatment, it was surprising that whether down-regulation of VEGF in ccRCC cells had any impact on tumor growth was not directly tested in a xenograft system. Besides it was discovered before that the tumors formed by 786-O cells were relatively insensitive to VEGF inhibitor sunitinib [34,35], so we decided to investigate whether the loss of VEGF expression would reduce tumor growth in 786-O-derived tumors. The positive result confirmed that VEGF expression was still important to tumor growth, but it is not as critical as HIF itself. The stronger effect of the shRNA than that of sunitinib could simply be due to the relative constant effect of shRNA while drug might fail to sufficiently inhibits its target between doses.

As CCND1 was a critical oncogene for breast cancer, we sought to address its importance in this model. As expected, it also positively contributed to the tumor growth but its efficacy was moderate compared to HIF.

Since HIF2α overexpression in *VHL+/+* 786-O cells was sufficient to generate big tumors in the xenograft model, we wondered whether the overexpression of an HRG could do the same. The overexpression of neither VEGF nor CCND1 in *VHL+/+* 786-O cells was able to cause robust tumor growth (data not shown). This was consistent with the observation from another lab (Othon Iliopoulos, personal communication). Thus it seems that although both VEGF and Cyclin D1 were able to promote tumor growth, neither of which was as powerful as HIF.

Although ANGPTL4 was reported to increase or decrease tumor growth and angiogenesis depending on the cancer types [24], it did not seem to be a major player in this model as its efficient suppression did not alter how fast the tumors grew. EGLN3 was found to be among the most highly induced HRGs, and it is known that it can biochemically hydroxylate key proline residues on the HIFα proteins [38] and its depletion led to HIF2α stabilization [37]. Since in 786-O cells the VHL protein is absent, this would not lead to the destruction of HIFα protein and did not obviously change the transcriptional activity of HIF. It is also known that EGLN3 has other hydroxylase targets [29,30] and have HIF-dependent and HIF-independent biological activities [31], so we wondered whether EGLN3 loss would have any impact on tumor growth. The *VHL-/-* 786-O cells depleted of EGLN3 grew tumors just as well as the control cells, suggesting that EGLN3 inhibition alone is not a good way to suppress kidney tumor growth. Similarly, suppression of ENO2 expression did not slow down the tumor growth. Either its function is not critical for tumor growth, or the remaining ENO1, or another activated compensatory pathway circumvents the loss of ENO2 protein.

It was totally unexpected that the suppression of GLUT1 enhanced, instead of suppressed, the tumor growth. This seemed to disagree with a report that a class of compound, exemplified by STF-31, could directly bind and inhibit GLUT1 function and selectively kill VHL-deficient RCC cancer cells [40]. It was further revealed that the STF-31 inhibited glucose uptake, significantly reduced ATP synthesis, led to cell death and inhibited tumor growth in xenograft models [40]. But a major difference exists between our studies: we only suppressed GLUT1 expression, while STF-31 potentially could inhibit all glucose transporter’s activity. Since GLUT1, GLUT2, GLUT3, GLUT4 are all expressed at different levels in RCC and they are all capable of transporting glucose [40], it is highly possible that in our system the loss of GLUT1 led to compensatory up-regulation of another glucose transporter which led to worse outcome, while STF-31 was able to inhibit the whole family of glucose transporters to inhibit tumor growth. Indeed in RCC4 *VHL+/+* cells where the GLUT1 expression was much lower than that in the RCC4 *VHL-/-* cells, the GLUT2 level in the *VHL+/+* cells was much higher than that in the *VHL-/-* cells, presumably due to a compensatory mechanism [40]. Whatever the explanation, our result indicates that the inhibition of GLUT1 alone is highly unlikely to be a successful therapeutic strategy against RCC, while the ability to inhibit all glucose transporters might be the key to achieve clinical efficacy.

Our previous finding that the suppression of JARID1C, an HRG, actually enhanced tumor growth suggested the existence of other tumor-inhibitory HRGs [41]. We found that the suppression of IGFBP3 very significantly increased the tumor growth, suggesting that it is another potent tumor-inhibitory HRG. As it binds to IGF which is an activator of IGFIR, a known oncogene in many types of human cancer cells [42], we investigated IGFIR activation status after IGFBP3 depletion. No obvious activation of IGFIR was discovered, suggesting its tumor-inhibitory activity might be IGFIR independent. We further investigated whether IGFIR suppression did reduce tumor growth, and the result confirmed that the expression of IGFIR was essential for efficient tumor growth. Surprisingly, loss of IGFIR increased both the mRNA level, and to a greater extent, the protein level of IGFBP3, further suggesting that the elevated IGFBP3 might be capable of suppressing tumor growth in the absence of IGFIR. As IGFBP3 were reported to interact with TGF-β pathway [43,44], increase the ratio of apoptotic (Bax and Bad) to the anti-apoptotic proteins (Bcl2 and Bcl-X_L_) [45], inhibit NF-κB activity [46], its abilities to inhibits cell growth/promotes apoptosis in an IGFIR-independent manner were well documented and might be at play here [33]. So IGFIR could be a very good drug target in addition to VEGFR in RCC, and any event that significantly enhances IGFBP3 expression might be desirable when treating RCC.

Our results indicate that there might be other HRGs that mediate the critical oncogenic activity of HIF in RCC, as VEGF or Cyclin D1 overexpression failed to drive efficient tumor growth in *VHL+/+* cells as the overexpression of HIF2α did. However, it is also possible that the combined effects of many tumor-promoting HRGs, including VEGF and Cyclin D1, were responsible for the tumor-inducing power of HIF (Figure 6F). It is an open question whether the overexpression of a single HRG is capable of driving efficient tumor growth in *VHL+/+* 786-O cells as HIF2α could. To further complicate the matter, the contributions of HIF-induced microRNAs might also be important as well, and further research might reveal whether one or more HRGs or microRNAs, in addition to VEGF and other important HRGs, might channel the major oncogenic activity of HIF that can be targeted for efficient therapeutic intervention in RCC. 

## Supporting Information

Table S1 **The list of the primers used for real-time PCR experiments in this paper.**
(DOCX)

File S1 **File includes Figures S1, S2, S3, and S4.** **Figure S1.** VHL status or change of HRG expression in 786-O cells does not alter *in vitro* growth rates. 786-O *VHL+/+*, 786-O *VHL-/-* cells (A), 786-O *VHL-/-* cells expressing either control shRNA (SCR) or VEGF-1137 (B), CCND1-2322 (C), ANGPTL4-786 (D), EGLN3-692 (E), ENO2-1992 (F), GLUT1-2310 (G), IGFBP3-711 (H) were used to compare *in vitro* proliferation rates. **Figure S2.** 786-O cells with the HIF1β depleted have diminished expression of the HRG GLUT1. 786-O *VHL-/-* cells expressing either SCR or HIF1b-1770 were either untreated or treated with hypoxia mimetics DFO or CoCl_2_ overnight. The cells were washed, lysed and subjected to western blots with indicated antibodies. **Figure S3.** The confirmation of HRGs in 786-O cells. Total RNAs were extracted from 786-O *VHL+/+*, 786-O *VHL-/-* cells, 786-O *VHL-/-* cells expressing two shRNA constructs against HIF2α, and 786-O *VHL+/+* cells expressing either a functional HIF2α mutant or a non-functional HIF2α mutant. First strand cDNA was generated from these samples then analyzed by real-time PCR for the indicated genes of interest. **Figure S4.** IGFBP3 suppression does not lead to increase of tyrosine phosphorylation on IGFIR in 786-O cells. Lysates from 786-O cell stably expressing either SCR or IGFBP3 shRNAs were used for immunoprecipitation of IGFIRβ. The immunoprecipitates were immunoblotted with indicated antibodies.
(TIF)

## References

[1] Linehan WM, Vasselli J, Srinivasan R, Walther MM, Merino M et al. (2004) Genetic basis of cancer of the kidney: disease-specific approaches to therapy. Clin Cancer Res 10: 6282S-6289S. doi:10.1158/1078-0432.CCR-050013. PubMed: 15448018. 15448018

[2] Kaelin WG Jr. (2002) Molecular basis of the VHL hereditary cancer syndrome. Nat Rev Cancer 2: 673-682. doi:10.1038/nrc885. PubMed: 12209156. 12209156

[3] Ivan M, Kondo K, Yang H, Kim W, Valiando J et al. (2001) HIFalpha targeted for VHL-mediated destruction by proline hydroxylation: implications for O2 sensing. Science 292: 464-468. doi:10.1126/science.1059817. PubMed: 11292862. 11292862

[4] Jaakkola P, Mole DR, Tian YM, Wilson MI, Gielbert J et al. (2001) Targeting of HIF-alpha to the von Hippel-Lindau ubiquitylation complex by O2-regulated prolyl hydroxylation. Science 292: 468-472. doi:10.1126/science.1059796. PubMed: 11292861. 11292861

[5] Yu F, White SB, Zhao Q, Lee FS (2001) HIF-1alpha binding to VHL is regulated by stimulus-sensitive proline hydroxylation. Proc Natl Acad Sci U S A 98: 9630-9635. doi:10.1073/pnas.181341498. PubMed: 11504942. 11504942 PMC55503

[6] Rini BI, Sosman JA, Motzer RJ (2005) Therapy targeted at vascular endothelial growth factor in metastatic renal cell carcinoma: biology, clinical results and future development. BJU Int 96: 286-290. doi:10.1111/j.1464-410X.2005.05616.x. PubMed: 16042715. 16042715

[7] Marangoni E, Vincent-Salomon A, Auger N, Degeorges A, Assayag F et al. (2007) A new model of patient tumor-derived breast cancer xenografts for preclinical assays. Clin Cancer Res 13: 3989-3998. doi:10.1158/1078-0432.CCR-07-0078. PubMed: 17606733. 17606733

[8] Motzer RJ, Hutson TE, Tomczak P, Michaelson MD, Bukowski RM et al. (2007) Sunitinib versus interferon alfa in metastatic renal-cell carcinoma. N Engl J Med 356: 115-124. doi:10.1056/NEJMoa065044. PubMed: 17215529. 17215529

[9] Rini BI, Atkins MB (2009) Resistance to targeted therapy in renal-cell carcinoma. Lancet Oncol 10: 992-1000. doi:10.1016/S1470-2045(09)70240-2. PubMed: 19796751. 19796751

[10] Escudier B, Eisen T, Stadler WM, Szczylik C, Oudard S et al. (2007) Sorafenib in advanced clear-cell renal-cell carcinoma. N Engl J Med 356: 125-134. doi:10.1056/NEJMoa060655. PubMed: 17215530. 17215530

[11] Sonpavde G, Hutson TE, Rini BI (2008) Axitinib for renal cell carcinoma. Expert Opin Investig Drugs 17: 741-748. doi:10.1517/13543784.17.5.741. PubMed: 18447599. 18447599

[12] Bukowski RM (2010) Pazopanib: a multikinase inhibitor with activity in advanced renal cell carcinoma. Expert Rev Anticancer Ther 10: 635-645. doi:10.1586/era.10.38. PubMed: 20469994. 20469994

[13] Kondo K, Klco J, Nakamura E, Lechpammer M, Kaelin WG Jr. (2002) Inhibition of HIF is necessary for tumor suppression by the von Hippel-Lindau protein. Cancer Cell 1: 237-246. doi:10.1016/S1535-6108(02)00043-0. PubMed: 12086860. 12086860

[14] Maranchie JK, Vasselli JR, Riss J, Bonifacino JS, Linehan WM et al. (2002) The contribution of VHL substrate binding and HIF1-alpha to the phenotype of VHL loss in renal cell carcinoma. Cancer Cell 1: 247-255. doi:10.1016/S1535-6108(02)00044-2. PubMed: 12086861. 12086861

[15] Kondo K, Kim WY, Lechpammer M, Kaelin WG Jr. (2003) Inhibition of HIF2alpha is sufficient to suppress pVHL-defective tumor growth. PLOS Biol 1: E83 PubMed: 14691554. 14691554 10.1371/journal.pbio.0000083 PMC300692

[16] Yan Q, Bartz S, Mao M, Li L, Kaelin WG Jr. (2007) The hypoxia-inducible factor 2alpha N-terminal and C-terminal transactivation domains cooperate to promote renal tumorigenesis in vivo. Mol Cell Biol 27: 2092-2102. doi:10.1128/MCB.01514-06. PubMed: 17220275. 17220275 PMC1820491

[17] Li L, Zhang L, Zhang X, Yan Q, Minamishima YA et al. (2007) Hypoxia-inducible factor linked to differential kidney cancer risk seen with type 2A and type 2B VHL mutations. Mol Cell Biol 27: 5381-5392. doi:10.1128/MCB.00282-07. PubMed: 17526729. 17526729 PMC1952077

[18] Tsao CC, Teh BT, Jonasch E, Shreiber-Agus N, Efstathiou E et al. (2008) Inhibition of Mxi1 suppresses HIF-2alpha-dependent renal cancer tumorigenesis. Cancer Biol Ther 7: 1619-1627. doi:10.4161/cbt.7.10.6583. PubMed: 19018165. 19018165

[19] Covello KL, Kehler J, Yu H, Gordan JD, Arsham AM et al. (2006) HIF-2alpha regulates Oct-4: effects of hypoxia on stem cell function, embryonic development, and tumor growth. Genes Dev 20: 557-570. doi:10.1101/gad.1399906. PubMed: 16510872. 16510872 PMC1410808

[20] Gunaratnam L, Morley M, Franovic A, de Paulsen N, Mekhail K et al. (2003) Hypoxia inducible factor activates the transforming growth factor-alpha/epidermal growth factor receptor growth stimulatory pathway in VHL(-/-) renal cell carcinoma cells. J Biol Chem 278: 44966-44974. doi:10.1074/jbc.M305502200. PubMed: 12944410. 12944410

[21] Wright TM, Brannon AR, Gordan JD, Mikels AJ, Mitchell C et al. (2009) Ror2, a developmentally regulated kinase, promotes tumor growth potential in renal cell carcinoma. Oncogene 28: 2513-2523. doi:10.1038/onc.2009.116. PubMed: 19448672. 19448672 PMC2771692

[22] Wright TM, Rathmell WK (2010) Identification of Ror2 as a hypoxia-inducible factor target in von Hippel-Lindau-associated renal cell carcinoma. J Biol Chem 285: 12916-12924. doi:10.1074/jbc.M109.073924. PubMed: 20185829. 20185829 PMC2857057

[23] Zimmer M, Doucette D, Siddiqui N, Iliopoulos O (2004) Inhibition of hypoxia-inducible factor is sufficient for growth suppression of VHL-/- tumors. Mol Cancer Res 2: 89-95. PubMed: 14985465. 14985465

[24] Tan MJ, Teo Z, Sng MK, Zhu P, Tan NS (2012) Emerging roles of angiopoietin-like 4 in human cancer. Mol Cancer Res 10: 677-688. doi:10.1158/1541-7786.MCR-11-0519. PubMed: 22661548. 22661548

[25] Yu Q, Geng Y, Sicinski P (2001) Specific protection against breast cancers by cyclin D1 ablation. Nature 411: 1017-1021. doi:10.1038/35082500. PubMed: 11429595. 11429595

[26] Raval RR, Lau KW, Tran MG, Sowter HM, Mandriota SJ et al. (2005) Contrasting properties of hypoxia-inducible factor 1 (HIF-1) and HIF-2 in von Hippel-Lindau-associated renal cell carcinoma. Mol Cell Biol 25: 5675-5686. doi:10.1128/MCB.25.13.5675-5686.2005. PubMed: 15964822. 15964822 PMC1157001

[27] Muller FL, Colla S, Aquilanti E, Manzo VE, Genovese G et al. (2012) Passenger deletions generate therapeutic vulnerabilities in cancer. Nature 488: 337-342. doi:10.1038/nature11331. PubMed: 22895339. 22895339 PMC3712624

[28] Kaelin WG Jr. (2011) Cancer and altered metabolism: potential importance of hypoxia-inducible factor and 2-oxoglutarate-dependent dioxygenases. Cold Spring Harb Symp Quant Biol 76: 335-345. doi:10.1101/sqb.2011.76.010975. PubMed: 22089927. 22089927 PMC4197849

[29] Schlisio S (2009) Neuronal apoptosis by prolyl hydroxylation: implication in nervous system tumours and the Warburg conundrum. J Cell Mol Med 13: 4104-4112. doi:10.1111/j.1582-4934.2009.00881.x. PubMed: 19691672. 19691672 PMC2847199

[30] Xie L, Xiao K, Whalen EJ, Forrester MT, Freeman RS et al. (2009) Oxygen-regulated beta(2)-adrenergic receptor hydroxylation by EGLN3 and ubiquitylation by pVHL. Sci Signal 2: ra33 PubMed: 19584355. 19584355 10.1126/scisignal.2000444 PMC2788937

[31] Su Y, Loos M, Giese N, Hines OJ, Diebold I et al. (2010) PHD3 regulates differentiation, tumour growth and angiogenesis in pancreatic cancer. Br J Cancer 103: 1571-1579. doi:10.1038/sj.bjc.6605936. PubMed: 20978507. 20978507 PMC2990580

[32] Adekola K, Rosen ST, Shanmugam M (2012) Glucose transporters in cancer metabolism. Curr Opin Oncol 24: 650-654. doi:10.1097/CCO.0b013e328356da72. PubMed: 22913968. 22913968 PMC6392426

[33] Jogie-Brahim S, Feldman D, Oh Y (2009) Unraveling insulin-like growth factor binding protein-3 actions in human disease. Endocr Rev 30: 417-437. doi:10.1210/er.2008-0028. PubMed: 19477944. 19477944 PMC2819737

[34] Huang D, Ding Y, Zhou M, Rini BI, Petillo D et al. (2010) Interleukin-8 mediates resistance to antiangiogenic agent sunitinib in renal cell carcinoma. Cancer Res 70: 1063-1071. doi:10.1158/0008-5472.CAN-09-3965. PubMed: 20103651. 20103651 PMC3719378

[35] Bhatt RS, Wang X, Zhang L, Collins MP, Signoretti S et al. (2010) Renal cancer resistance to antiangiogenic therapy is delayed by restoration of angiostatic signaling. Mol Cancer Ther 9: 2793-2802. doi:10.1158/1535-7163.MCT-10-0477. PubMed: 20699227. 20699227 PMC2956167

[36] Pestell RG (2013) New roles of cyclin d1. Am J Pathol 183: 3-9. doi:10.1016/S0002-9440(13)00360-X. PubMed: 23790801. 23790801 PMC3702737

[37] Taniguchi CM, Finger EC, Krieg AJ, Wu C, Diep AN et al. (2013) Cross-talk between hypoxia and insulin signaling through Phd3 regulates hepatic glucose and lipid metabolism and ameliorates diabetes. Nat Med, 19: 1325–30. PubMed: 24037093. 24037093 10.1038/nm.3294 PMC4089950

[38] Epstein AC, Gleadle JM, McNeill LA, Hewitson KS, O'Rourke J et al. (2001) C. elegans EGL-9 and mammalian homologs define a family of dioxygenases that regulate HIF by prolyl hydroxylation. Cell 107: 43-54. doi:10.1016/S0092-8674(01)00507-4. PubMed: 11595184. 11595184

[39] Zhang H, Gao P, Fukuda R, Kumar G, Krishnamachary B et al. (2007) HIF-1 inhibits mitochondrial biogenesis and cellular respiration in VHL-deficient renal cell carcinoma by repression of C-MYC activity. Cancer Cell 11: 407-420. doi:10.1016/j.ccr.2007.04.001. PubMed: 17482131. 17482131

[40] Chan DA, Sutphin PD, Nguyen P, Turcotte S, Lai EW et al. (2011) Targeting GLUT1 and the Warburg effect in renal cell carcinoma by chemical synthetic lethality. Sci Transl Med 3: 94ra70 PubMed: 21813754. 10.1126/scitranslmed.3002394 PMC3683134 21813754

[41] Niu X, Zhang T, Liao L, Zhou L, Lindner DJ et al. (2012) The von Hippel-Lindau tumor suppressor protein regulates gene expression and tumor growth through histone demethylase JARID1C. Oncogene 31: 776-786. doi:10.1038/onc.2011.266. PubMed: 21725364. 21725364 PMC4238297

[42] Boone DN, Lee AV (2012) Targeting the insulin-like growth factor receptor: developing biomarkers from gene expression profiling. Crit Rev Oncog 17: 161-173. doi:10.1615/CritRevOncog.v17.i2.30. PubMed: 22471706. 22471706 PMC3926653

[43] Oh Y, Müller HL, Lamson G, Rosenfeld RG (1993) Insulin-like growth factor (IGF)-independent action of IGF-binding protein-3 in Hs578T human breast cancer cells. Cell surface binding and growth inhibition. J Biol Chem 268: 14964-14971. PubMed: 7686909. 7686909

[44] Oh Y, Müller HL, Pham H, Rosenfeld RG (1993) Demonstration of receptors for insulin-like growth factor binding protein-3 on Hs578T human breast cancer cells. J Biol Chem 268: 26045-26048. PubMed: 7504671. 7504671

[45] Butt AJ, Firth SM, King MA, Baxter RC (2000) Insulin-like growth factor-binding protein-3 modulates expression of Bax and Bcl-2 and potentiates p53-independent radiation-induced apoptosis in human breast cancer cells. J Biol Chem 275: 39174-39181. doi:10.1074/jbc.M908888199. PubMed: 10998426. 10998426

[46] H-Zadeh AM, Collard TJ, Malik K, Hicks DJ, Paraskeva C et al. (2006) Induction of apoptosis by the 16-kDa amino-terminal fragment of the insulin-like growth factor binding protein 3 in human colonic carcinoma cells. Int J Oncol 29: 1279-1286. PubMed: 17016662. 17016662

