# Supplementary material for: The Contributions of HIF-Target Genes to Tumor Growth in RCC
Source: PLoS One. 2013 Nov 18;8(11):e80544. doi: 10.1371/journal.pone.0080544 (PMC3832366; doi:10.1371/journal.pone.0080544)
Supplement: Table S1 — The list of the primers used for real-time PCR experiments in this paper. (DOCX) [file pone.0080544.s001.docx]

**Supplemental TABLE 1:**

|  | |  |  |
| --- | --- | --- | --- |
|  | |  |  |
| Table 1 Primers For Real time PCR | | | |
|  | Forward | Reverse |  |
| IGFBP3 | CAGAGCACAGATACCCAGAAC | AGCACATTGAGGAACTTCAGG |  |
| GLUT1 | TCATCGTGGCTGAACTCTTC | GATGAAGACGTAGGGACCAC |  |
| CCND1 | CATCTACACCGACAACTCCATC | TCTGGCATTTTGGAGAGGAAG |  |
| VEGFA | GGCAGCTTGAGTTAAACGAAC | AGCGTGGTTTCTGTATCGATC |  |
| ACTIN | ATCGTCCACCGCAAATGCTTCTA | AGCCATGCCAATCTCATCTTGTT |  |
| CXCR4 | GCTTGCTGAATTGGAAGTGAATG | TCTTCACGGAAACAGGGTTC |  |
| EGLN3 | ATT CAT AGC AGA TGT GGA GCC | TCAGCATCAAAGTACCAGACAG |  |
| ENO2  ANGPTL4  IGFIR  HIF2A  HIG2  CA12  MTUS1  ZNF395  CP  MXI-1  ARRDC3  SERPINE1  APOL1  DDIT4  DUSP1 | GGCACTCTACCAGGACTTTG  AGACACAACTCAAGGCTCAG  CATACCTCAACGCCAATAAGT  CCCATGTCTCCACCTTCAAG  TGAGTTTTGTGGCGGGAAG  CAGGTCCAGAAGTTCGATGAG  CCAAGTTTGAGGCATTGACAG  TTCTCCCCAAACTGATCATGG  CTGGTATCTGATGGGAATGGG  CTCAGGAGATGGAACGAATACG  TCTTGCCTCCACCTCTTTATTC  GACAGACCCTTCCTCTTTGTG  GCGGACCAAGAACTGTGAC  GTTCGCACACCCATTCAAG  ACCACAAGGCAGACATCAG | GTTTTGGGTTGGTCACTGTC  CTCATGGTCTAGGTGCTTGTG  TATCTCGCGTCATACCAAA  GGCTTGCTCTTCATACTCCAG  GCTGCCTTCTCCTTCTGAAAG  CAATACAGATGCCAAGAATGCC  TGGTTGAAGCAGTGACTAGC  CAATGGAGCGCAGAACTTTG  CTAGGGTTTGGTATGTTCCAGG  ATGGGAGAACTCTGTGCTTTC  TCGGAACCCACATCAACTTG  TCTCCAGTTTTGTCCCAGATG  CACCATTGCACTCCAACTTG  GTCCCAAAGGCTAGGCATG  AAGGTAAGCAAGGCAGATGG |  |
