# Supplementary figures and images for: The Contributions of HIF-Target Genes to Tumor Growth in RCC
Source: PLoS One. 2013 Nov 18;8(11):e80544. doi: 10.1371/journal.pone.0080544 (PMC3832366; doi:10.1371/journal.pone.0080544)

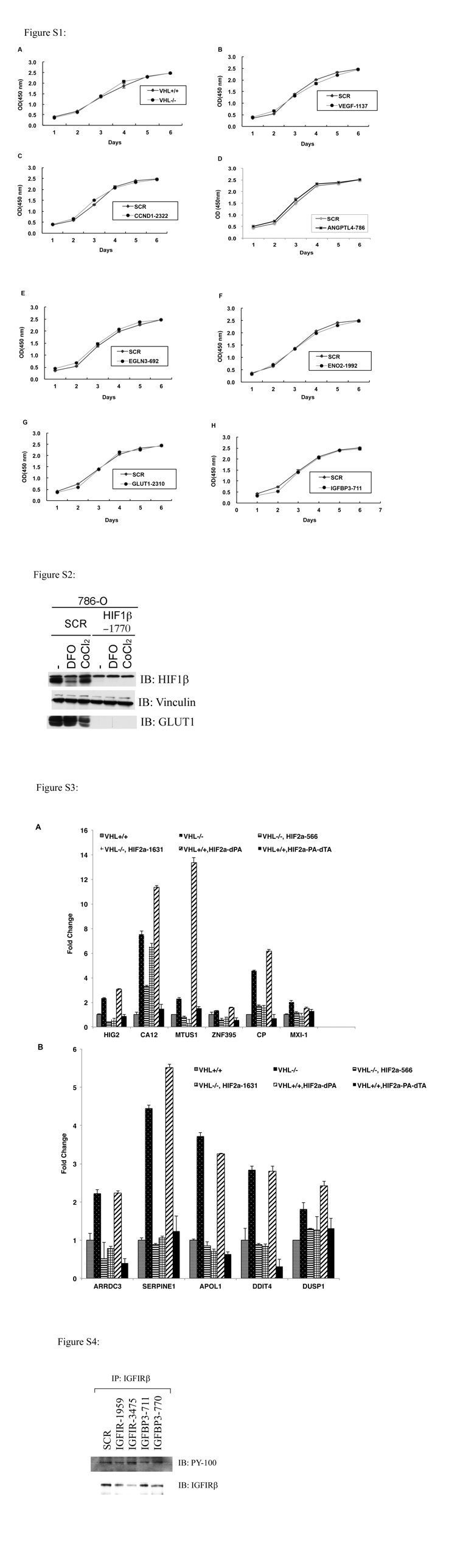

Supplement: File S1 — File includes Figures S1, S2, S3, and S4. Figure S1. VHL status or change of HRG expression in 786-O cells does not alter in vitro growth rates. 786-O VHL+/+, 786-O VHL-/- cells (A), 786-O VHL-/- cells expressing either control shRNA (SCR) or VEGF-1137 (B), CCND1-2322 (C), ANGPTL4-786 (D), EGLN3-692 (E), ENO2-1992 (F), GLUT1-2310 (G), IGFBP3-711 (H) were used to compare in vitro proliferation rates. Figure S2. 786-O cells with the HIF1β depleted have diminished expression of the HRG GLUT1. 786-O VHL-/- cells expressing either SCR or HIF1b-1770 were either untreated or treated with hypoxia mimetics DFO or CoCl2 overnight. The cells were washed, lysed and subjected to western blots with indicated antibodies. Figure S3. The confirmation of HRGs in 786-O cells. Total RNAs were extracted from 786-O VHL+/+, 786-O VHL-/- cells, 786-O VHL-/- cells expressing two shRNA constructs against HIF2α, and 786-O VHL+/+ cells expressing either a functional HIF2α mutant or a non-functional HIF2α mutant. First strand cDNA was generated from these samples then analyzed by real-time PCR for the indicated genes of interest. Figure S4. IGFBP3 suppression does not lead to increase of tyrosine phosphorylation on IGFIR in 786-O cells. Lysates from 786-O cell stably expressing either SCR or IGFBP3 shRNAs were used for immunoprecipitation of IGFIRβ. The immunoprecipitates were immunoblotted with indicated antibodies. (TIF) [file pone.0080544.s002.tif]
